# Isoflavone Containing Legumes Mitigate Ergot Alkaloid-Induced Vasoconstriction in Goats (*Capra hircus*)

**DOI:** 10.3390/ani12060750

**Published:** 2022-03-17

**Authors:** Brittany E. Harlow, Michael D. Flythe, Jack P. Goodman, Huihua Ji, Glen E. Aiken

**Affiliations:** 1 Forage-Animal Production Research Unit, USDA-ARS, Lexington, KY 40506, USA; michael.flythe@usda.gov (M.D.F.); gaiken@ufl.edu (G.E.A.); 2 Plant and Soil Sciences Department, University of Kentucky, Lexington, KY 40546, USA; jpgood2@email.uky.edu; 3 Kentucky Tobacco Research and Development Center, University of Kentucky, Lexington, KY 40546, USA; hji4@email.uky.edu

**Keywords:** ergot alkaloids, goats, isoflavones, tall fescue, vasoconstriction

## Abstract

**Simple Summary:**

When endophyte-infected tall fescue is consumed by grazing livestock it can cause persistent vasoconstriction resulting in fescue toxicosis. Legumes are commonly utilized in livestock production to improve diet quality. Legumes also contain vasodilatory isoflavones that have been shown to alleviate fescue toxicosis in goats and grazing cattle. Different legumes contain varying levels and types of isoflavones. A pen study was conducted to determine if isoflavone supplementation via red clover, white clover, or soybean meal can mitigate vasoconstriction associated with fescue toxicosis in goats. Rumen fistulated Wether goats were assigned to each legume treatment at equal levels of supplementation by weight. Goats were subjected to a fescue toxicosis challenge with toxic tall fescue seed, and the carotid artery area was monitored using Doppler ultrasonography. All isoflavone treatments were able to partially mitigate vasoconstriction. Red clover, with the greatest concentration of isoflavones, was the most effective. These results demonstrate that red clover, white clover, and soybean meal supplementation can be used to reverse the vasoconstriction associated with fescue toxicosis in goats despite differences in isoflavone concentration and composition. The impact of this research is a legume-derived phytochemical that can be applied in ruminants consuming toxic tall fescue to reverse fescue toxicosis and improve animal health and productivity.

**Abstract:**

Ergot alkaloids produced by a fungal endophyte that infects tall fescue (*Lolium arundinaceum*; (E+ TF) can induce constriction of the vasculature in ruminants, resulting in “fescue toxicosis”. Legumes contain isoflavones that have been demonstrated to prevent and reverse E+ TF vasoconstriction. Several legumes are conventionally utilized in ruminant production, but can vary in both isoflavone concentration and composition. A feeding study was conducted to determine if isoflavone supplementation via red clover (*Trifolium pratense*), white clover (*Trifolium repens*), or soybean (*Glycine max*) meal can alleviate vasoconstriction when wether goats were challenged with E+ TF seed. The basal diet was chopped grass hay ad libitum. Carotid luminal areas were obtained pre- and post-ruminal infusions of E+ TF seed (15 µg kg BW^−1^ ergovaline + ergovalanine ± red clover, white clover, or soybean meal at 2.61 mg kg BW^−1^). When goats were challenged with E+ TF seed, the mean carotid luminal areas decreased by 56.1% (*p* < 0.01). All treatments were able to partially mitigate vasoconstriction, with red clover being the most effective (+39.8%), and white clover and soybean meal eliciting an intermediate response (+30%, *p* < 0.01). Results indicate that legumes can relax vasoconstriction in goats consuming ergot alkaloids, despite differences in isoflavone profile and concentrations.

## 1. Introduction

Tall fescue (*Lolium arundinaceum*) is the most prevalent cool-season perennial grass in the United States, covering approximately 14 million ha [1]. The vast majority of tall fescue is infected by a fungal endophyte (*Epichloë coenophiala*) that produces alkaloids allowing tall fescue to be persistent and productive over a wide range of environments [2,3,4]. However, the endophyte also produces ergot alkaloid toxins (e.g., ergovaline; [5]) that binds biogenic amine receptors in the vascular system causing persistent vasoconstriction [6], and consequently incapacitating the animal’s ability to thermoregulate [7]. Grazing livestock suffering from “fescue toxicosis” often have reduced weight gain and reproductive performance, heat tolerance and serum prolactin [8,9,10].

Many plants, including forage species in pastures, can produce antimicrobial secondary metabolites that improve rumen fermentation and function [11,12,13,14]. Legumes are conventionally incorporated into livestock production systems to improve the diet quality and diversity. Legumes also produce isoflavones (most notably Biochanin A) that selectively inhibit bacteria in the rumen that waste dietary protein, promote bacteria that utilize fiber, and consequently improve average daily gains of grazing cattle [15,16]. Therefore, targeted isoflavone supplementation has been identified as an alternative and effective antimicrobial growth promoter. After absorption, isoflavones have phytoestrogenic activity that have been shown to cause arterial vasodilation via nitric oxide synthase activation [17,18,19,20]. In a study by Aiken et al. [21], isoflavone supplementation was demonstrated to relax arteries and reduce resistance of blood flow when goats were challenged with endophyte-infected tall fescue (E+ TF) seed. Similarly, interseeding red clover (*Trifolium pratense*) in E+ TF pastures has been demonstrated to alleviate vasoconstriction in grazing steers [22]. However, similar experiments with other legumes as isoflavone sources were never attempted.

Legumes, including red and white clover, are commonly interseeded in grazing systems and soybean (as meal or hulls) are commonly supplemented to grazing ruminants to improve intake and nutrition. However, these legumes vary in both isoflavone concentration and composition [23,24,25,26]. Therefore, a feeding study was conducted to determine if isoflavone supplementation via red clover, white clover (*Trifolium repens*), or soybean (*Glycine max*) meal can mediate relaxation of the carotid artery during ergot-alkaloid-induced vasoconstriction in wether goats.

## 2. Materials and Methods

The experiment was conducted in indoor facilities on the University of Kentucky campus using pens in a room maintained at a temperature of approximately 20 °C. All husbandry and procedures were approved by the Institutional Animal Care and Use Committee at the University of Kentucky. General housing and care of the animals were consistent with the Guide to Care and Use of Agricultural Animals in Research and Teaching [27].

### 2.1. Experimental Design

Spanish, rumen-fistulated mature wether goats (*n* = 12) were used in the experiment. Initial body weights averaged 46.3 ± 3.5 kg and final body weights averaged 44.8 ± 3.3 kg. Due to the limited number of fistulated goats available, the experiment was conducted utilizing a 3 × 3 crossover design to allow for an increased statistical power. Goats were paired by body weight, to minimize competition for feed, and assigned to six pens with two goats per pen. The goats were first adapted to their pens and fed a basal diet of chopped orchardgrass (*Dactylis glomerata*; also called cocksfoot)—timothy (*Phleum pratense*) hay ad libitum for two weeks (Table 1). The goats were then subjected to three back-to-back experimental periods consisting of a 1 week E+ tall fescue seed challenge followed by a 1 week treatment period. Goats were blocked by body weight for treatment assignment (four goats block^−^^1^, three total blocks).

During the E+ tall fescue seed challenge periods, goats were dosed trans-ruminally with ground (2.0-mm screen; Wiley Mill, Thomas Scientific, Swedesboro, NJ, USA) E+ tall fescue seed daily at a rate of 15 µg kg BW^−^^1^ ergovaline + ergovalanine. This dose of ergovaline + ergovalanine provided as ground seed introduced via a ruminal cannula has been previously reported to sufficiently induce fescue toxicosis (reduced intake, increased respiration rates, vasoconstriction, etc.) in cattle [28,29,30]. The ground seed used was from the endophyte-infected tall fescue cultivar, “Defender”. Prior to the start of the experiment, seed was analyzed for concentrations of the ergopeptine, ergovaline, and its epimer, ergovalinine, using the procedures of Yates and Powell [31] and modified as described by Carter et al. [32]. Concentrations of ergovaline/ergovalinine in the seed averaged 3.76 μg g seed^−^^1^.

At the start of each treatment period, seed dosing was continued, and blocks were randomly assigned to one of three treatments: (1) red clover, (2) white clover, or (3) soybean meal. The red clover (Kenland var.) and white clover (Alice var.) treatments were harvested fresh from established plots and oven dried (60 °C in a forced air-drying oven). The soybean meal was obtained from a commercial source. All legume treatments were ground (2.0-mm screen; Wiley Mill; Thomas Scientific, Swedesboro, NJ, USA) prior to dosing. The quantity of red clover trans-ruminally dosed daily (2.61 g kg BW^−^^1^) was set to match the infusion rate used in Aiken and colleagues [21] of 30 mg L^−^^1^ Biochanin A. The white clover and soybean meal treatments were dosed daily at the same rate on a total mass basis (2.61 g kg BW^−^^1^).

The experimental period was repeated three times to allow for each block to receive all three treatments. Feeding of hay and rumen infusions were given at 09.00 h each day. Intake of hay in each pen was monitored and recorded daily, and the goats were weighed weekly to inform infusion rates. All goats were provided loose minerals and water ad libitum over the course of the experiment.

### 2.2. Color Doppler-Ultrasonography

Images of the left carotid artery cross-sections were collected via Color Doppler ultrasound using a Classic Medical TeraVet 3000 Ultrasound Unit (Classic Universal Ultrasound, Tequesta, FL, USA) with a 12 MHz linear array transducer (12L5-VET). Ultrasound scans were collected on the last 2 days of the adaptation period to provide a baseline measurement. Scans were then taken on the last 2 days of each E+ tall fescue challenge period and treatment period. All ultrasound sessions started at approximately 14:00 h and were completed within 1 h. Prior to the start of the study, the goats were acclimated to regular handling to reduce excitability. The hair of each goat was clipped at the imaging site with surgical clippers prior to the adaptation period and at the start of each experimental period. Cross-sectional images (4 cm depth) were collected using a frequency of 5.0 MHz and a pulse repetitive frequency between 2.5 and 3.0 kHz. From each collected scan, the ultrasound images exhibiting maximum flow signal (peak systolic phase) were traced to estimate lumen area [33].

### 2.3. Isoflavone Analyses

Prior to the start of the study, the red clover, white clover, and soybean meal were analyzed for isoflavone concentrations using the method described in Aiken et al. [21] and modified as follows (Table 2). Feed samples were ground through a 1-mm mesh in a Wiley mill. Tissue (100 to 250 mg) was extracted (85% methanol containing 0.5% aqueous acetic acid) by sonicating (30 min; model 5510 sonicating water bath, Bransonics Corporation, Danbury, CT, USA). Water (3 mL) was added to the extracts, to achieve a final solvent composition of 60% methanol in 0.35% acetic acid. The mixture was centrifuged (2200× *g*, 25 °C) for 8 min and the resulting supernatant was filtered through a 0.45-μm GHP hydrophilic membrane (Pall Corporation, Port Washington, NY, USA).

Isoflavone extracts were analyzed by LC-MS (Waters Acquity UPLC coupled to a Waters Synapt G2 (q-ToF) high resolution mass spectrometer; Waters Corporation, Milford, MA, USA). The high-resolution mass spectrometer was operated in positive ion electrospray mode (resolving power of ~14,000) and scanned from 100 to 1000 Da in 0.3 s. Chromatographic separation was obtained using a Waters BEH C18 UPLC column (1.7 μm, 2.1 mm × 150 mm). The mobile phase employed a mixture of water containing 0.1% formic acid and acetonitrile containing 0.1% formic acid in a linear gradient from 20% B to 80% B (flow rate: 0.35 mL/min). Leucine enkephalin was used to provide a lock mass (*m*/*z* 554.2615). Quantification of isoflavones was performed with a linear calibration curve and flavone as the internal standard (QuanLynx Software; Waters Corporation). Extracted ion chromatograms (mass window ±0.02 Da around the accurate mass) of each analyte were used to calculate peak areas. To quantify malonyl glucosides, each sample was analyzed as-extracted and a second portion was heated at 85 °C for 5 h to hydrolyze the isoflavone malonyl-glucosides to their corresponding glucosides. Concentrations of biochanin A malonyl-glucoside and formononetin-malonyl-glucoside were determined by the difference between the hydrolyzed and un-hydrolyzed portions.

### 2.4. Statistical Analyses

Measures from images collected at the end of the adaptation period were averaged for each goat (average baseline measure), subtracted from measures made in each period, and then divided by the average baseline measure to estimate the proportionate difference of luminal areas of the carotid artery. All data were analyzed using the MIXED procedure of SAS (SAS v. 9.3; SAS Inst. Inc., Cary, NC, USA). The model for the proportionate difference data included animal as the experimental unit, proportionate difference as the dependent variable and treatment (E+ TF control, red clover, white clover, soybean meal), block, period, and the interaction of treatment × period as fixed effects. To evaluate the effect of treatment on the feed intake, pen intake data (% pen BW hay intake, DM basis) was compared statistically on each day Ultrasound measures were collected (last 2 days of each E+ tall fescue challenge period and treatment period). Due to treatments being assigned to individual animals as opposed to by pen, intake comparisons could only be made between E+ TF control and legume treatment. Therefore, the model for the intake data included pen as the experimental unit, feed intake as the dependent variable and treatment (E+ TF control, legume treatment), period, and the interaction of treatment × period as fixed effects. In all analyses, the Kenward–Roger method was used to compute the denominator degrees of freedom for the fixed effects. When a main effect was detected, means were separated using the pdiff option. Statistical significance was set at *p* < 0.05.

## 3. Results

**Figure 1:**
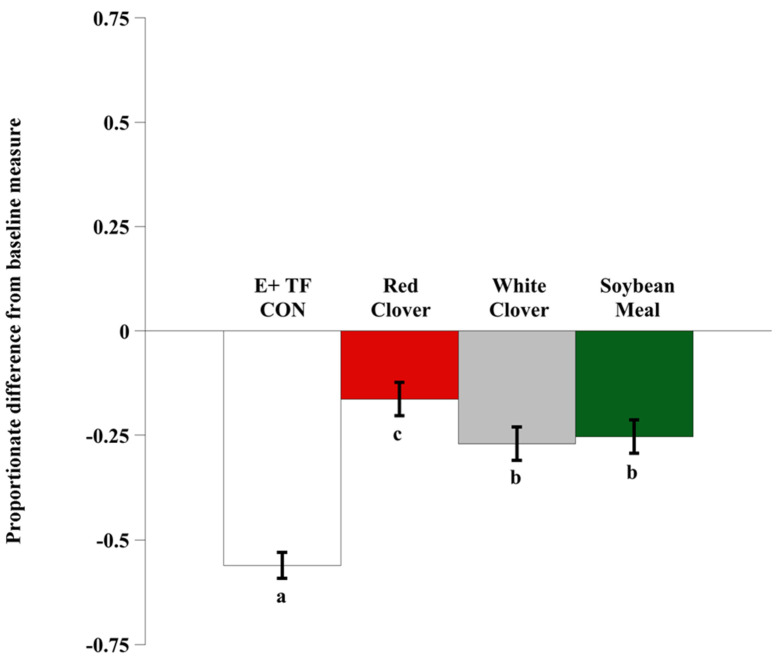
Ultrasonic measures of proportionate differences from baseline measures for luminal area of the carotid artery in rumen-fistulated wether goats that were infused with toxic E+ TF seed ± red clover, white clover, or soybean meal. Baseline measures were taken with goats receiving ad libitum chopped orchardgrass (*Dactylis glomerata*)—timothy (*Phleum pratense*) hay with no rumen infusion treatments. Images for determining baseline measures were collected on the last 2-d of the adaptation period. Means lacking a common letter (a,b,c) are different. Treatment: *p* < 0.0001; period: *p* = 0.2415; block: *p* = 0.3030; period × treatment: *p* = 0.8491; Pooled SEM: Treatment = 0.0313; period = 0.0354; block = 0.0532; period × treatment = 0.0639.

Baseline measures of the carotid luminal areas averaged 23.7 ± 6.0 mm^2^. There was no effect of block (*p* = 0.3030), period (*p* = 0.2415), or treatment × period interaction (*p* = 0.8491) detected in the proportionate differences of carotid artery luminal areas (Figure 1). However, there was an effect of isoflavone treatment (*p* < 0.0001). When E+ TF seed (E+ TF CON) was infused in the rumen, the mean carotid luminal area decreased an average of 56.1% relative to the hay-only baseline (*p* < 0.01). The carotid luminal area remained less than the baseline, regardless of treatment, but all isoflavone treatments were able to reduce vasoconstriction relative to the vasoconstricted control (E+ TF CON). Red clover treatment was the most effective improving vasoconstriction, 39.8% relative to the E+ TF CON measures (*p* < 0.01). Both white clover and soybean meal provided a similar intermediate effect of alleviating vasoconstriction by 29.1% (*p* < 0.01) and 30.7% (*p* < 0.01), respectively, relative to E+ TF CON.

On average, goats in the current study consumed 2.04 ± 0.03% BW as hay DM at the end of the adaptation period. There was no effect of period (*p* = 0.1495) or treatment × period interaction (*p* = 0.7223) detected on pen hay intake. However, there was an effect of isoflavone treatment (*p* < 0.0342). When E+ TF seed was infused trans-ruminally, pen hay intake decreased to 1.08 ± 0.03% BW DM^−^^1^. However, the addition of legume treatment increased pen hay intake 39% relative to E+ TF CON (1.61 ± 0.06% BW DM^−^^1^; *p* < 0.05).

## 4. Discussion

The objective of this study was to determine the efficacy of different legume sources (red clover, white clover, and soybean meal) in mitigating vasoconstriction in animals undergoing fescue toxicosis. Ergot alkaloids produced by toxic tall fescue bind adrenergic and serotonergic receptors in the vascular system to cause persistent constriction of blood flow to peripheral tissues [34]. The resulting peripheral vasoconstriction disrupts thermoregulation causing the animal to be more susceptible to severe cold or heat stress, even under moderate ambient air temperatures [35,36,37]. Recent research has also indicated that ergot alkaloids induce vasoconstriction of the ruminal artery, ruminal vein, and mesenteric vasculature in cattle which could negatively impact nutrient efficiencies [38,39]. Therefore, strategies to alleviate vasoconstriction in ruminants suffering from fescue toxicosis are needed.

Interseeding toxic endophyte-infected tall fescue pastures with clovers or supplementing other legume-based feeds (e.g., soyhulls, soybean meal), has long been recommended to mitigate performance losses and the negative health impacts of fescue toxicosis in grazing ruminants [22,32,40,41,42,43]. Historically, the benefits of legume addition have primarily been attributed to the improvement of diet intake and the quality or ‘dilution’ reducing total consumption of ergot alkaloids [44,45,46,47]. In addition to their nutritional benefits, legumes contain phytoestrogenic isoflavones that have been shown to have impacts on the vasculature by activating nitric oxide synthase arterial vasodilation [48]. In human medicine, isoflavones have been used as a treatment for ameliorating hot flashes in postmenopausal women [17,18], reducing hypertension [49], and decreasing the risk of coronary heart disease [20,50]. Similarly, Shappell and colleagues [43] observed an additive effect of combining soybean hulls and estradiol implants on increasing the estrogenic activity of serum and mitigating fescue toxicosis in grazing steers [32]. Although the mechanism was not elucidated, the authors hypothesized that the observed mitigation of fescue toxicosis could have been attributed to the vaso-active isoflavones present in the soybean hulls. Aiken and colleagues [21] confirmed this hypothesis by demonstrating that isoflavone supplementation could both reverse and prevent vasoconstriction of the carotid and interosseous arteries in goats challenged with E+ TF seed. Most recently, our research group demonstrated that interseeding red clover in E+ TF pastures alleviated vasoconstriction and improved growth performance in grazing steers [22].

In the current study, the E+ TF seed challenge successfully induced fescue toxicosis vasoconstriction as evidenced by a 56% reduction in carotid artery areas relative to the hay-only baseline. The level of vasoconstriction observed was similar to reports by Aiken et al. [21] of a 45% reduction in goat carotid artery areas with E+ TF seed challenge. Treatment with all three legumes reversed vasoconstriction, with red clover being most effective (+40% relative to E+ TF seed control). Soybean meal and white clover both produced a similar intermediate response (+30% relative to E+ TF seed control). Isoflavone treatment also improved hay intake in comparison to the E+ TF CON. Although individual legume treatment differences could not be evaluated due to experimental design constraints, these results might suggest that the alleviation of vasoconstriction observed could be a ‘dilution’ response. However, research by Beck and colleagues [51] reported similar improvements in grazing steers’ performance when clovers were added to either toxic or non-toxic TF pastures, disproving the ‘dilution’ hypothesis. Therefore, the increased hay intake with legume addition is likely to be in response to alleviation of vasoconstriction, as opposed to being causative.

The legume source-dependent response observed could be attributed to the variable concentration and composition of isoflavones present. Red clover and white clover both contain predominantly biochanin A and formononetin, with lower levels of genistein and daidzein present [19,25,26]. However, these clovers are also different in their total isoflavone content. The white clover utilized in this experiment contained 0.05% total isoflavones, but the red clover material contained 1.3% total isoflavones (24× higher). In contrast to the clovers, soybean meal contains predominantly daidzein and genistein and an intermediate level of total isoflavones [23,24]. The soybean meal utilized in the current study contained 0.13% total isoflavones, 10× less total isoflavones than those present in the red clover treatment. The red clover amount in the current study matched the previous study [21]. We considered including all legumes at an amount that would deliver equal total isoflavones. However, to deliver equal isoflavones in white clover or soy would have exceeded the volumes of the goat rumens. Instead, we chose to give all legumes on an equal weight basis, more closely reflecting realistic consumption of each feed type.

Another consideration unique to ruminants is the fermentative conversion of isoflavones in the rumen prior to absorption. Jia and colleagues [52] were not able to detect any vasoactive impacts of the isoflavones’ formononetin or biochanin A in bovine mesenteric arteries and veins exposed to E+ TF seed extract in vitro. This is consistent with reports that the vaso-activity of isoflavone metabolites may be greater than the pure isoflavones themselves [19,53]. In the rumen, resident microorganisms demethylate formononetin to daidzein and biochanin A to genistein. Daidzein can then be converted to equol or 0-desmethylangolensin and genistein converted to p-ethyl phenol [54,55]. Equol has been demonstrated to have an order of magnitude higher estrogenic activity than its precursor [56]. In contrast, p-ethyl phenol has no estrogenic activity [57]. Therefore, it is possible that the concentration of specific isoflavone precursors like formononetin and daidzein might inform vasodilatory potential. For example, red clover treatment in the current study elicited the greatest vasodilation and had 11× greater concentrations of formononetin and daidzein than white clover and soybean meal. The white clover and soybean meal treatments had relatively similar combined concentrations of formononetin and daidzein and elicited equal vasoactive benefits. Future research is needed to evaluate the significance of isoflavone composition, bioavailability, and metabolism on vasodilatory responses in ruminants.

## 5. Conclusions

The results of this study demonstrate that red clover, white clover, and soybean meal supplementation can mitigate ergot alkaloid-induced vasoconstriction and feed intake depression in goats, despite vast differences in isoflavone concentration and composition. Additional experiments are required to determine how isoflavone composition influences vasoactive potential and to identify threshold concentrations of isoflavones in the diet to yield maximum benefits.

Decreased serum prolactin is commonly utilized as an indicator of fescue toxicosis. Although it would have been interesting to monitor serum prolactin in the current study, blood collection would have impacted on temperament and heart rate and thus cause extraneous error in the ultrasound measures. Future research is needed to evaluate the impact of different isoflavone sources and delivery methods on physiological and phenotypic indicators of fescue toxicosis in grazing ruminants.

## Figures and Tables

**Table 1:** Nutrient composition of chopped orchardgrass (*Dactylis glomerata)*—timothy (*Phleum pratense*) hay, red clover, white clover, and soybean meal (dry matter basis) ^1^.

Nutrient ^2^	Hay	Red Clover	White Clover	Soybean Meal
DM, %	89.41	88.13	88.84	86.36
CP, %	8.89	15.46	17.34	46.14
ADF, %	38.12	32.32	29.64	6.84
NDF, %	65.81	40.21	38.11	14.36
IVTD, %	60.19	72.61	76.32	82.14

^1^ Basal diet: Hay ad libitum; Treatments: 2.61 g kg BW^−1^ red clover, white clover, or soybean meal; ^2^ DM—dry matter; CP—crude protein; ADF—acid detergent fiber; NDF—neutral detergent fiber; IVTD—in vitro true digestibility.

**Table 2:** Isoflavone concentrations in red clover, white clover, and soybean meal.

Isoflavone (µg g DM^−1^)	Red Clover	White Clover	Soybean Meal
Biochanin A	1979.35	8.05	14.61
Biochanin A Glucoside (sissotrin)	696.39	2.55	0.00
Biochanin A Malonyl-Glucoside	3295.44	24.50	0.00
Formononetin	2580.05	176.45	7.52
Formononetin Glucoside (ononin)	659.94	26.74	0.90
Formononetin Malonyl-Glucoside	3252.64	284.99	0.00
Genistein	102.75	11.82	31.57
Genistein Glucoside (genistin)	124.02	1.67	622.38
Daidzein	106.84	3.32	20.15
Daidzein Glucoside (daidzin)	287.96	2.90	592.94

## Data Availability

All data are reported in the manuscript text.
